# Enhanced survival prediction using explainable artificial intelligence in heart transplantation

**DOI:** 10.1038/s41598-022-23817-2

**Published:** 2022-11-14

**Authors:** Paulo J. G. Lisboa, Manoj Jayabalan, Sandra Ortega-Martorell, Ivan Olier, Dennis Medved, Johan Nilsson

**Affiliations:** 1 Department of Applied Mathematics, Liverpool John Moores University, Liverpool, UK; 2 Department of Translational Medicine, Artificial Intelligence and Bioinformatics in Cardiothoracic Sciences, Lund University, Lund, Sweden; 3 Department of Thoracic and Vascular Surgery, Skane University Hospital, Lund, Sweden

**Keywords:** Cardiology, Risk factors, Outcomes research, Machine learning

## Abstract

The most limiting factor in heart transplantation is the lack of donor organs. With enhanced prediction of outcome, it may be possible to increase the life-years from the organs that become available. Applications of machine learning to tabular data, typical of clinical decision support, pose the practical question of interpretation, which has technical and potential ethical implications. In particular, there is an issue of principle about the predictability of complex data and whether this is inherent in the data or strongly dependent on the choice of machine learning model, leading to the so-called accuracy-interpretability trade-off. We model 1-year mortality in heart transplantation data with a self-explaining neural network, which is benchmarked against a deep learning model on the same development data, in an external validation study with two data sets: (1) UNOS transplants in 2017–2018 (n = 4750) for which the self-explaining and deep learning models are comparable in their AUROC 0.628 [0.602,0.654] *cf.* 0.635 [0.609,0.662] and (2) Scandinavian transplants during 1997–2018 (n = 2293), showing good calibration with AUROCs of 0.626 [0.588,0.665] and 0.634 [0.570, 0.698], respectively, with and without missing data (n = 982). This shows that for tabular data, predictive models can be transparent and capture important nonlinearities, retaining full predictive performance.

## Introduction

Heart transplantation is life-saving for patients with end-stage heart disease. A limiting factor is the lack of donor organs. Recently, the United Network for Organ Sharing (UNOS) organ allocation system has been changed to a more granular distinction of medical urgency, resulting in lower waitlist mortality. However, recent analysis has shown worse posttransplantation survival^1^. To reduce posttransplantation mortality, the factors affecting posttransplantation survival must be better understood^2^.

Artificial intelligence is experiencing explosive growth in clinical applications from specialist data processing, such as radiology and text mining, to diagnostic and prognostic risk stratification with tabular data typically comprised of clinical factors, demographics and multiple indicators derived from physiological measurements. This opens the potential for more precise predictions that add to the evidence base used for clinical decision making. Since the value of quantitative outcome models is highest where data are complex and noisy, this raises the question of a trade-off between model complexity and predictive performance, particularly in real-world settings with high stake outcomes. Recent studies in organ transplantation have shown that machine learning (ML) techniques have the potential to improve outcomes for transplant recipients. However, work is needed to improve the interpretation of these algorithms, ensure generalizability through external validation, and create an infrastructure to enable clinical integration^3^.

This study is about the prioritisation of patients to receive a scarce and highly valuable resource, a donor's heart. For the clinician, the first priority is to have access to the most accurate predictive model. Linked to this is the ethical question of justifying the choice of the recipient based on all of the available evidence, now augmented with an AI-based prediction.

A recent review indicates that there is no appreciable performance loss for sparse, linear-in the-parameters models compared with deep learning applied to tabular data, that is to say flat, unstructured data with a mixture of largely independent continuous and discrete variables, widely used in clinical decision making^4^. This is especially the case when the signal-to-noise ratio is low. This finding echoes the view that machine learning models for high stake decisions should be interpretable by design as part of a drive to encourage responsible machine learning governance^5^. Model transparency brings additional advantages when seeking to find and fix problems in data sets, such as control of bias, which is a key consideration for observational data frequently used in cross-sectional and cohort studies^6^.

Among the algorithmic challenges for interpretable machine learning, generalized additive models (GAMs) are attractive, as they are inherently interpretable, certainly to the same level of understanding as popular risk stratification algorithms such as logistic regression^5^. Additive models have generic value for interpretability^7^ and meet accepted criteria for robust interpretability laid out in the following desiderata^8^—explicitness/intelligibility: *“Are the explanations immediate and understandable?”;* faithfulness: “*Are relevance scores indicative of "true" importance?”;* and stability: “*How consistent are the explanations for similar/neighbouring examples?”*. In addition, GAMs can also achieve parsimony/sparsity *“Do the explanatory variables comprise a minimal set?”* and consistency: *“How robust are the explanations to perturbations in the data?”.*

The challenge is to maintain the well-known data fitting capabilities of neural networks without sacrificing predictive performance^9^.

The main novel contribution of the paper is to show, with a substantive external validation study, that this question can be addressed by a method to efficiently derive a self-explaining neural network model while preserving the predictive performance of a much larger deep learning model. We derive an accurate predictor in the form of a sparse model with meaningful features constructed through an iterative process. The method is generic for tabular data in real-world applications where non-linear effects are estimated in the presence of significant levels of noise.

The motivation for the Partial Response Network (PRN) is that multivariate functions can be written as a summation of functions of fewer variables^6,10^. In our paper this is done in a novel way by utilising the formal framework of functional Analysis of Variance (ANOVA) decompositions where the component functions have interesting theoretical properties^11^. In particular, they are orthogonal with respect to a given metric, which provides a principled way to separate univariate from bivariate terms. Since our component functions are identified from a trained neural network, we can proceed to model selection without having to also estimate the terms of the GAM in parallel. If the metric is a Dirac function, then the component functions in the ANOVA decomposition are derived from cuts of the multivariate function at a given point, which we choose to be the overall median of the data. This is known as an anchored ANOVA decomposition. In application to medical data, the key principle is that the derived functions of one or two variables represent main effects and pairwise interactions that, between them, can reasonably comprise much of the discriminant information that is required of the model. The methodology to derive and optimize these functions is explained in more detail in the “Methods” section.

The first novel contribution of this paper is to benchmark the classification performance of the PRN model with that of a previously published deep learning model (International Heart Transplant Survival Algorithm [IHTSA]) and a traditional scoring model (Index for Mortality Prediction After Cardiac Transplantation [IMPACT])^12,13^. This study aims to assess the relative performance of the interpretable model and a deep neural network with two external cohorts, one comprising recent heart transplants from the UNOS data and the other with transplant data from a regional database in Scandinavia. This is a gold standard benchmarking study since it uses three data cohorts: 1997–2013 to train the model and 2014–2016 to find the hyperparameters that optimise model performance, but a subsequent cohort of patients operated on in 2017–2018 to calculate the generalisation performance and compare it between the different models.

The second novelty is to verify consistency between the independent effects identified by the PRN for individual variables and pairs of variables and clinical expertise. Our study seeks to move the focus of discussion of machine learning models of clinical risk from mainly classification performance, where different models often have comparable performance, to clinical insights coupled with performance, meaning interpretability without compromising predictive accuracy.

A further benchmark included for completeness is to compare against an alternative interpretable method from the machine learning literature, Explainable Boosting Machines (EBMs)^14^. In common with the PRN, this uses the structure of a GAM. However, it models the univariate and bivariate component functions using rule ensembles, resulting in staggered rather than smooth functions. Moreover, there is no constraint of orthogonality between the component functions, as is the case in the ANOVA decomposition that underpins the PRN. The model results are compared in the “Discussion” section.

## Results

### Patient demographics in all cohorts/population

**Figure 1:**
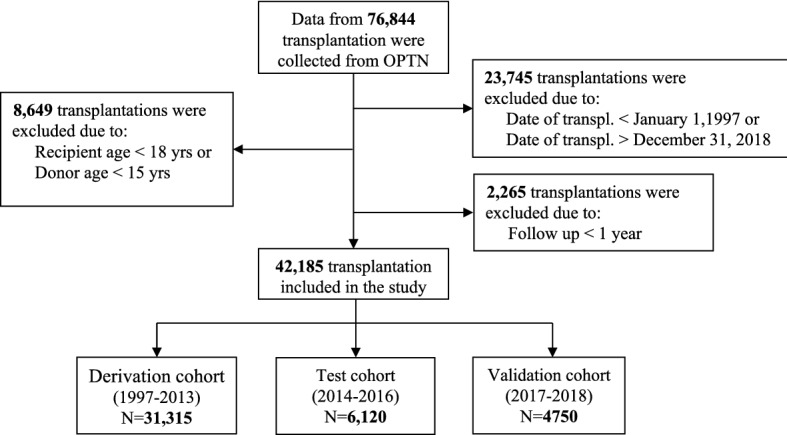
Flow chart. Flow diagram for recipients collected from OPTN. OTPN denotes Organ Procurement and Transplantation Network.

Clinically relevant information about transplanted patients collected from the UNOS database between 1997 and 2016 was used for model development (derivation cohort [DC], n = 31,315) and test cohort ([TC], n = 6120). The third cohort of patients transplanted between 2017 and 2018 was used as an external blinded validation cohort (VC, n = 4750) (Fig. 1). The derivation and validation cohorts comprised 296,451 patient years (median survival time 12.1 years, IQR 5.3–19.2). The 1-year mortality was 11.8% (n = 4978). The three study cohorts, DC, TC, and the blinded external VC, have a different distribution of most of the included variables. As shown in Tables 1, 2, the patients were older, heavier, and presented with more comorbidities, such as diabetes mellitus and need for dialysis, in the VC group than in the DC group. Furthermore, the presence of critical state variables such as ECMO, inotropic support, and mechanical assistance were more common in VC. Despite this, the 1-year mortality was significantly lower in the VC (9.8%) compared with TC (10.8%) and DC (12.3%), p < 0.001 (Kruskal–Wallis test). On the other hand, the duration of donor heart ischemia, infection, and need for a ventilator before transplantation were lower in the VC group than in the DC group.

**Table 1:** Recipient baseline characteristics for the study cohorts.

	N	Ext. validation 2017–2018 *N* = 4750	Test 2014–2016 N = 6120	Train/validation 1997–2013 N = 31,315	Test statistic
Age (yrs)*^,#,†^	42,185	47 57 64 54 ± 13	47 56 63 54 ± 13	46 55 61 52 ± 12	*F*_2 42182_ = 91, P < 0.001^2^
Female gender*^,†^	42,185	26%^1244^⁄_4750_	26%^1562^⁄_6120_	24%^7423^⁄_31,315_	χ^2^_2_ = 20, P < 0.001^1^
Height (cm)*	42,076	167.6 175.0 180.3 173.8 ± 10.1	167.6 175.0 180.3 173.8 ± 10.0	167.6 175.2 180.3 173.9 ± 9.7	*F*_2 42073_ = 1.7, P = 0.19^2^
Weight (kg)*	42,091	71 84 97 84 ± 18	70 83 95 83 ± 18	69 80 92 81 ± 17	*F*_2 42088_ = 109, P < 0.001^2^
**Race**^***#***^	42,185				χ^2^_8_ = 377, P < 0.001^1^
Asian		3.6%^169^⁄_4750_	3.6%^222^⁄_6120_	2.3%^709^⁄_31,315_	
Black		22.8%^1082^⁄_4750_	22.5%^1374^⁄_6120_	16.5%^5168^⁄_31,315_	
Hispanic		8.8%^420^⁄_4750_	8.7%^532^⁄_6120_	6.8%^2115^⁄_31,315_	
Other		1.2%^57^⁄_4750_	1.3%^78^⁄_6120_	1.0%^310^⁄_31,315_	
White		63.6%^3022^⁄_4750_	64.0%^3914^⁄_6120_	73.5%^23,013^⁄_31,315_	
Diagnosis*^,#,†^	42,185				χ^2^_10_ = 531, P < 0.001^1^
Congenital		3.1%^145^⁄_4750_	2.8%^171^⁄_6120_	2.4%^739^⁄_31,315_	
Graft failure		2.7%^126^⁄_4750_	2.8%^171^⁄_6120_	3.1%^975^⁄_31,315_	
ICM		30.7%^1460^⁄_4750_	34.2%^2094^⁄_6120_	43.8%^13,711^⁄_31,315_	
NICM		60.1%^2856^⁄_4750_	57.0%^3488^⁄_6120_	46.7%^14,616^⁄_31,315_	
Other		2.3%^111^⁄_4750_	1.9%^118^⁄_6120_	2.0%^617^⁄_31,315_	
Valve		1.1%^52^⁄_4750_	1.3%^78^⁄_6120_	2.1%^657^⁄_31,315_	
Diabetes mellitus*	41,700	29%^1382^⁄_4748_	29%^1780^⁄_6114_	24%^7313^⁄_30,838_	χ^2^_2_ = 124, P < 0.001^1^
Infection within 2 weeks*^,#,†^	42,181	9.1%^430^⁄_4750_	10.8%^662^⁄_6120_	10.7%^3343^⁄_31,311_	χ^2^_2_ = 12, P = 0.002^1^
Dialysis^#^	41,359	4.7%^222^⁄_4748_	4.2%^256^⁄_6117_	3.3%^1020^⁄_30,494_	χ^2^_2_ = 27, P < 0.001^1^
Previous blood transfusion*	39,870	21%^1009^⁄_4732_	27%^1598^⁄_5993_	22%^6387^⁄_29,145_	χ^2^_2_ = 69, P < 0.001^1^
Previous organ transpl.*	42,185	3.0%^142^⁄_4750_	3.0%^182^⁄_6120_	3.4%^1078^⁄_31,315_	χ^2^_2_ = 5.4, P = 0.069^1^
Prior cardiac surgery (non-OHT)*	29,165	50%^2380^⁄_4750_	56%^3416^⁄_6120_	48%^8727^⁄_18,295_	χ^2^_2_ = 121, P < 0.001^1^
Medical condition at OHT*	42,185				χ^2^_4_ = 78, P < 0.001^1^
Home		53%^2516^⁄_4750_	54%^3296^⁄_6120_	49%^15305^⁄_31,315_	
Hospital		16%^759^⁄_4750_	17%^1050^⁄_6120_	18%^5478^⁄_31,315_	
ICU		31%^1475^⁄_4750_	29%^1774^⁄_6120_	34%^10532^⁄_31,315_	
Ventilator at OHT*^,#,†^	42,185	0.9%^42^⁄_4750_	0.9%^58^⁄_6120_	2.6%^807^⁄_31,315_	χ^2^_2_ = 105, P < 0.001^1^
ECMO*	42,185	1.3%^64^⁄_4750_	0.8%^50^⁄_6120_	0.5%^150^⁄_31,315_	χ^2^_2_ = 54, P < 0.001^1^
IABP*^,#^	42,185	8.7%^414^⁄_4750_	7.0%^428^⁄_6120_	5.3%^1667^⁄_31,315_	χ^2^_2_ = 99, P < 0.001^1^
VAD at OHT*^,#^	36,831				χ^2^_10_ = 3317, P < 0.001^1^
LVAD		45.6%^2168^⁄_4750_	45.5%^2787^⁄_6120_	19.4%^5032^⁄_25,961_	
LVAD + RVAD		1.5%^71^⁄_4750_	2.0%^121^⁄_6120_	2.4%^625^⁄_25,961_	
No device		51.9%^2463^⁄_4750_	50.7%^3102^⁄_6120_	68.5%^17,772^⁄_25,961_	
RVAD		0.2%^11^⁄_4750_	0.2%^10^⁄_6120_	0.2%^54^⁄_25,961_	
TAH		0.8%^37^⁄_4750_	1.6%^100^⁄_6120_	0.8%^196^⁄_25,961_	
Unknown device		0.0%^0^⁄_4750_	0.0%^0^⁄_6120_	8.8%^2282^⁄_25,961_	
PVR (wood units)*	34,075	1.4 2.0 2.8 2.3 ± 1.4	1.4 2.0 3.0 2.4 ± 1.5	1.4 2.1 3.1 2.5 ± 1.7	*F*_2 34072_ = 23, P < 0.001^2^
SPP (mmHg)*	39,300	29 37 47 39 ± 13	30 38 49 40 ± 14	32 41 52 42 ± 14	*F*_2 39297_ = 197, P < 0.001^2^
Creatinine (μmol/l)*^,#,†^	41,367	85 106 133 120 ± 74	85 106 133 119 ± 69	88 106 133 120 ± 68	*F*_2 41364_ = 4.8, P = 0.008^2^
Serum bilirubin (μmol/l)*^,#^	40,640	6.8 12.0 17.1 16.6 ± 31.8	8.6 12.0 18.8 17.0 ± 33.1	8.6 13.7 22.2 20.9 ± 41.6	*F*_2 40637_ = 313, P < 0.001^2^
PRA > 10%*	36,978	24%^916^⁄_3773_	22%^1149^⁄_5287_	15%^4188^⁄_27,918_	χ^2^_2_ = 306, P < 0.001^1^
HLA-DR mismatch*	27,383				χ^2^_4_ = 18, P = 0.001^1^
0		4.6%^156^⁄_3410_	5.2%^226^⁄_4312_	4.2%^830^⁄_19,661_	
1		37.6%^1283^⁄_3410_	38.1%^1642^⁄_4312_	40.1%^7883^⁄_19,661_	
2		57.8%^1971^⁄_3410_	56.7%^2444^⁄_4312_	55.7%^10,948^⁄_19,661_	
ABO blood type*	42,185				χ^2^_6_ = 36, P < 0.001^1^
A		40.1%^1903^⁄_4750_	38.9%^2382^⁄_6120_	42.2%^13,222^⁄_31,315_	
AB		5.2%^248^⁄_4750_	6.0%^369^⁄_6120_	5.3%^1661^⁄_31,315_	
B		15.4%^730^⁄_4750_	15.4%^943^⁄_6120_	13.9%^4352^⁄_31,315_	
O		39.3%^1869^⁄_4750_	39.6%^2426^⁄_6120_	38.6%^12,080^⁄_31,315_	

a b c represents the lower quartile a, the median b, and the upper quartile c for continuous variables. x ± s represents X ± 1 SD. N is the number of non-missing values. Variables included in * IHTSA, ^#^ IMPACT, and ^†^ PRN model. SPP, Systolic pulmonary pressure; PRA, panel reactive antibody level, HLA, human leucocyte antigen; OHT, orthotopic heart transplantation; VAD, ventricular assist device; LVAD, left ventricular assist device; RVAD, Right ventricular assist device; TAH, total artificial heart; ECMO, extracorporeal membrane oxygenation. ICU, Intensive care unit; NICM, non-ischemic cardiomyopathy; ICM, ischemic cardiomyopathy; PVR, pulmonary vascular resistance; IABP, intra aortic balloon pump.

Tests used: (1) Pearson test; (2) Kruskal–Wallis test.

**Table 2:** Donor baseline characteristics for the study cohorts.

	N	Ext. validation 2017–2018 *N* = 4750	Test 2014–2016 *N* = 6120	Train/val. 1987–2013 *N* = 31,315	Test statistic
Donor age (yrs)*^,†^	42,185	24 31 40 33 ± 11	23 31 41 32 ± 11	21 30 42 32 ± 12	*F*_2 42182_ = 18, P < 0.001^2^
Donor female gender*	42,185	30%^1443^⁄_4750_	30%^1828^⁄_6120_	29%^9214^⁄_31,315_	χ^2^_2_ = 2.1, P = 0.36^1^
Donor height (cm)*	42,144	168.0 175.0 180.3 174.0 ± 9.6	167.6 175.0 180.3 173.8 ± 9.6	167.6 175.3 182.9 174.6 ± 9.5	*F*_2 42141_ = 19, P < 0.001^2^
Donor weight (kg)*	42,179	70 81 95 84 ± 19	70 80 93 83 ± 20	68 79 90 81 ± 18	*F*_2 42176_ = 69, P < 0.001^2^
Donor ABO blood type*	42,184				χ^2^_6_ = 7.7, P = 0.26^1^
A		36.7%^1745^⁄_4750_	35.3%^2163^⁄_6120_	36.4%^11,413^⁄_31,314_	
AB		2.1%^99^⁄_4750_	2.5%^154^⁄_6120_	2.3%^706^⁄_31,314_	
B		10.9%^520^⁄_4750_	11.3%^690^⁄_6120_	10.5%^3295^⁄_31,314_	
O		50.2%^2386^⁄_4750_	50.9%^3113^⁄_6120_	50.8%^15,900^⁄_31,314_	
Donor cause of death*	42,149				χ^2^_8_ = 2669, P < 0.001^1^
Anoxia		38.2%^1813^⁄_4748_	29.8%^1823^⁄_6118_	12.3%^3850^⁄_31,283_	
Cerebrovascular/stroke		14.8%^703^⁄_4748_	19.1%^1166^⁄_6118_	25.6%^8000^⁄_31,283_	
CNS tumor		0.5%^23^⁄_4748_	0.3%^21^⁄_6118_	1.0%^313^⁄_31,283_	
Head trauma		44.6%^2119^⁄_4748_	48.6%^2971^⁄_6118_	59.3%^18,544^⁄_31,283_	
Other		1.9%^90^⁄_4748_	2.2%^137^⁄_6118_	1.8%^576^⁄_31,283_	
Ischemic time (min)*^,†^	40,394	138 184 223 183 ± 64	143 186 227 187 ± 62	146 188 229 190 ± 63	*F*_2 40391_ = 23, P < 0.001^2^
Recipient-donor height ratio*	42,037	0.963 1.000 1.036 1.000 ± 0.056	0.967 1.000 1.040 1.002 ± 0.057	0.958 1.000 1.036 0.998 ± 0.061	*F*_2 42034_ = 16, P < 0.001^2^
Recipient-donor weight ratio*	42,086	0.87 1.03 1.18 1.03 ± 0.23	0.88 1.02 1.17 1.03 ± 0.22	0.88 1.02 1.16 1.02 ± 0.21	*F*_2 42083_ = 2.6, P = 0.071^2^

*a* *b* *c* represents the lower quartile *a*, the median *b*, and the upper quartile *c* for continuous variables. *x* ± *s* represents X ± 1 SD. *N* is the number of non-missing values. Variables included in * IHTSA, ^#^ IMPACT, and ^†^ PRN model. CNS, Central nervous system tumours. Tests used: ^1^Pearson test; ^2^Kruskal–Wallis test.

The starting pool of inputs for model selection by the PRN consists of variables commonly used to predict 1-year mortality (recipient age, donor age, creatinine, ischemic time, ventilator at orthotopic heart transplantation (OHT), diagnosis of nonischemic disease in recipients, recipient female sex, infection within 2 weeks, history of prior transplantation).

Supplementary Figs. 1, 2, 3, 4 shows the deviance and values of beta for both Lasso models, one derived from the original MLP and the other from the PRN model derived from it. The duration of ischemia came in early on as part of a core set of five variables (donor age, ischemic time, creatinine, ventilator at OHT, transplant year) selected even for very high regularisation. This indicates that the duration of ischemia, together with the other variables above, is consistently important.

**Figure 2:**
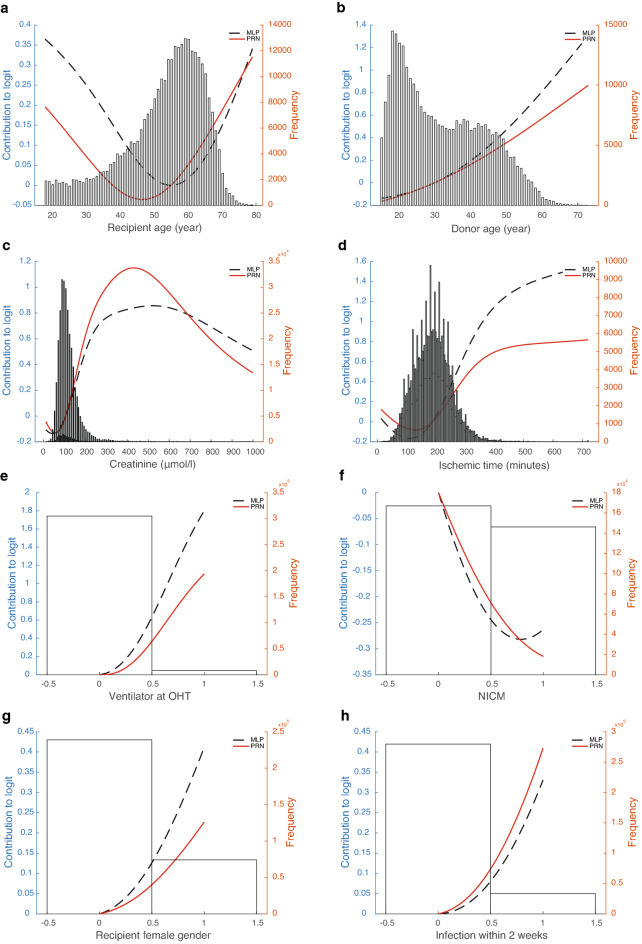

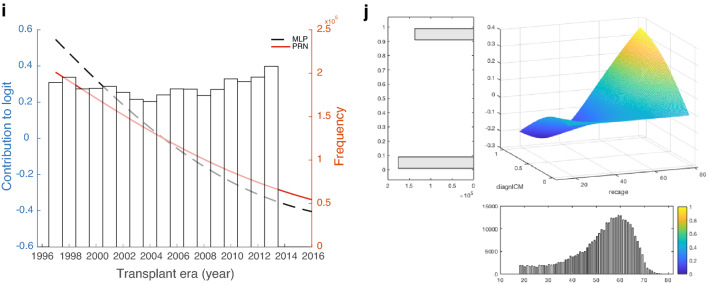
Partial response representing the additive contribution to the logarithm of the odds of death in year one. The partial response representing the additive contribution of: (**a**) recipient age (≥ 18 yrs), (**b**) donor age (≥ 15 yrs), (**c**) creatinine, (**d**) ischemic time, (**e**) ventilator at OHT, (**f**) diagnosis: nonischemic cardiomyopathy, (**g**) recipient female gender, (**h**) infection within 2 weeks, and (**i**) transplantation era to the logarithm of the odds of death in year one i.e. the logit. The black line shows the initial estimate of the partial response directly from the ANOVA decomposition, and the red curve is the same function recalibrated in the second step of back error propagation. Overlaid in the figure is the histogram of the input variable. (**j**) shows the partial response from the bivariate effect between recipient age and diagnosis of ICM. MLP denotes the partial responses obtained from the original neural network; PRN denotes the partial responses obtained after subsequent retraining of the SENN in Fig. 6.

The final PRN-Lasso model includes nine univariate effects: recipient age, donor age, creatinine, ischemic time, ventilator at OHT, diagnosis of nonischemic disease in recipients, recipient female sex, infection within 2 weeks, and transplant year, together with a 2-way interaction involving recipient age and diagnosis of ischemic cardiomyopathy (ICM) in recipients. The AUROC for the prediction of 1-year mortality was 0.653 (95% CI 0.643–0.662) in DC and 0.605 (95% CI 0.582–0.628) in TC. The partial response additive contribution to the logarithm of the odds of death in year one for each univariate effect is presented in Fig. 2a–i. As illustrated in Fig. 2a, the recipient age contributes to the model most at younger and older ages, so it has a U-shape, whereas the donor age contribution is monotonically increasing and close to linear (Fig. 2b). Figure 2c identifies a critical boundary for the influence of Creatinine on the 1-year mortality risk. It is expected that unusually high values of this variable are associated with an exceptionally high contribution to the mortality risk. The sparsity of data means that there will be a larger confidence interval associated with the risk estimate for each individual arising from this variable at very high values. The duration of ischemia starts to contribute significantly after three and a half hours (Fig. 2d). Figure 2e–h show functions that are evaluated only at binary values. Therefore, they in effect calculated odds-ratios for the least populated binary values of the variables against their median values. Figure 2i shows the effect of transplant era.

The 2-way interaction is of interest, as it suggests a protective effect for ischemic disease in younger recipients, in contrast to the expected increase in mortality for older age. A crude calculation of the hazard ratio for 1-year mortality by filtering recipients in the age groups 18–40 and 60–70 shows values of 0.117 and 0.124, respectively, lower than and marginally higher than the overall prevalence of 0.123 in the DC. This is consistent with the 2-way effect shown in Fig. 2j, which was found to be statistically significant by Lasso.

### Predictive performance

**Figure 3:**
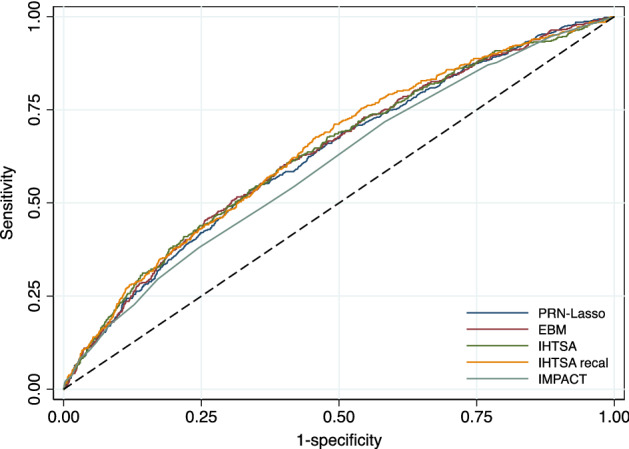
ROC curves of the different models. The ROC curves show the sensitivity and 1-specificity of the prediction of 1-year mortality for the PRN with 10 responses (navy blue solid line), EBM (maroon solid line), IHTSA (forest green solid line), IHTSA recalibrated (dark orange solid line) and IMPACT (teal green solid line) risk algorithms. The black dashed line represents the absence of discrimination. Heart transplant patients in the blinded validation cohort 2017–2018 (n = 4570) were included in the analysis.

**Figure 4:**
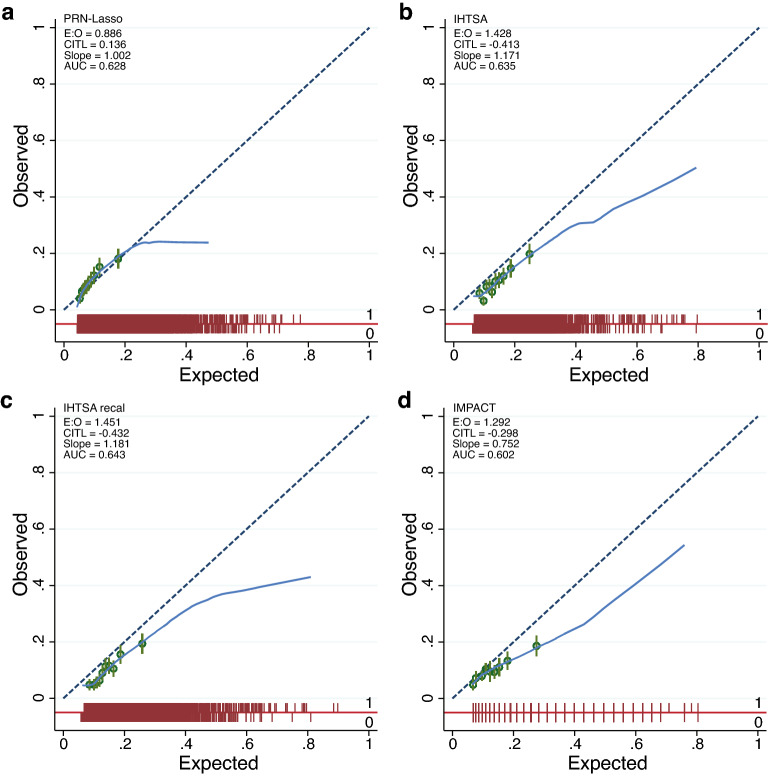
Calibration plot of PRN. The calibration plot, (**a**–**d**), shows observed against expected probabilities for assessment of the four prediction models with the overall external validation cohort according to the TRIPOD guidelines. The red spike plot shows the distribution of events and nonevents. Green circles indicate the expected probabilities in groups with 95% CI. The green line demonstrates the lower smoothness smoother. The model performance statistics are summarized with EO: exp/obs ratio; CITL: calibration-in-the-large; and AUC: area under the curve.

As shown in Table 3 and Fig. 3, the discrimination (AUROC) for the PRN-Lasso including 10 partial responses was similar for the IHTSA and IHTSA recalibrated models in the blinded external VC, 0.628 (CI 95%: 0.602–0.654) vs 0.635 (CI 95%: 0.609–0.662), p = 0.488, and 0.643 (CI 95%: 0.617–0.669), p = 0.197. The Hosmer–Lemeshow (HL) chi-square in VC was 15.01 for the PRN model (p = 0.135), suggesting that there was good calibration (Fig. 4a). As shown in Fig. 4b–d, the calibration for the IHTSA and IMPACT models was poor (p < 0.001). The discrimination for the IHTSA and IHTSA recalibrated models compared with the IMPACT model was significantly superior, p = 0.023 and p = 0.004, respectively.

**Table 3:** AUROC—exernal validation cohort.

	N	ROC area	[95% conf.]	P
PRN-Lasso	4750	0.628	0.602–0.654	
EBM^13^	4750	0.634	0.607–0.660	0.173
IHTSA^10^	4750	0.635	0.609–0.662	0.488
IHTSA recalibrated^26^	4750	0.643	0.617–0.669	0.197
IMPACT^11^	4750	0.602	0.575–0.628	0.094

PRN, partial response network; EBM, explainable boosting machines; IHTSA, International Heart Transplant Survival Algorithm; IMPACT, index for mortality prediction after cardiac transplantation.

The machine learning benchmarking method applied to the external cohort 2017–18, EBM, has a high estimated ROC area of 0.634 (CI 95%: 0.607–0.660), selecting the following variables: donor age, ischemic time, recipient age, creatinine, infection within 2 weeks, ventilator at OHT, female gender of the recipient, transplant era and diagnosis of ICM. These variables are entirely consistent with those selected by the PRN, lacking only the interaction between the diagnosis of ICM and NICM.

Further external validation of the PRN-LASSO model was carried out using a regional data set from the Scandia Thoracic Transplantation Database from Scandinavia (n = 2293). This time, the timeframe overlaps as the transplants took place over the period 1997–2018. There is also a significant proportion of missing data in all but 982 rows of data. Missing values were imputed using the median value for that column, as in data standardization, this maps onto zero so that missing values do not contribute to the model prediction. The AUROC for the full data set imputed was 0.626 (CI 95%: 0.588–0.665), and for observed values, it was only 0.634 (CI 95%: 0.570–0.698). It is of interest to note that the calibration of the model developed with UNOS data, when applied to the regional data, is almost perfect (Supplementary Figs. 5 and 6).

### Test cases in the external validation cohort

**Figure 5:**
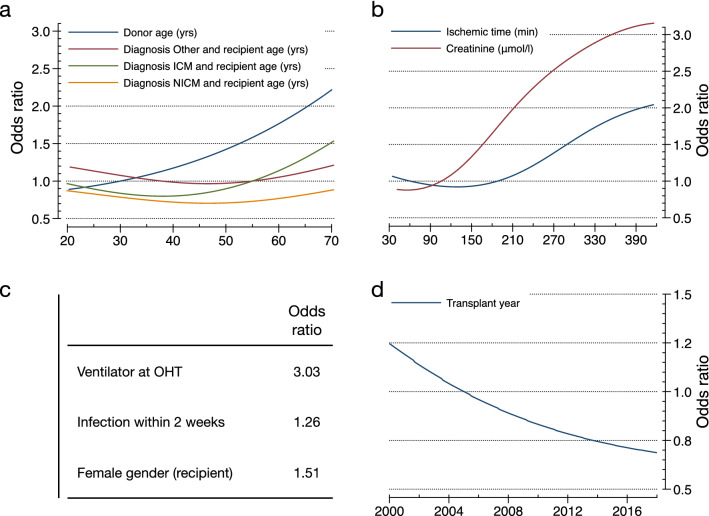
Nomogram predicting 1-year mortality after heart transplantation. The partial response representing the additive contribution: (**a**) donor age (blue solid line), recipient age and diagnosis Other (red solid line), recipient age and diagnosis ICM (green solid line), recipient age and diagnosis NICM (orange solid line); (**b**) creatinine (red solid line), and ischemic time (blue solid line), (**c**) ventilator at OHT, recipient female gender, infection within 2 weeks; (**d**) transplantation year to the odds of death in year one. To calculate the logarithm of the total odds for an individual patient, the logarithm of the odds ratios (contributing logits) are summed for all the contributing factors, adding an overall intercept of − 2.3354.

The self-explaining neural network has a modular form that, for each individual row of data, calculates separate univariate or bivariate functions. These functions are then added to form the logit of the model prediction. In the case of the GAMs, the component functions are first orthogonalized using an ANOVA decomposition. In both cases, the form of Equation 1 shows that the logit of the prediction is the sum of component functions, which we call partial responses, which are explicitly shown in Fig. 2. For an individual prediction, the input values for the variables in the functions in Fig. 2 generate contributions that are then added to make the logit that, in turn, is put through the sigmoid function to predict the 1-year mortality prediction. A detailed example for a particular patient is shown in Supplementary Tables 1 and 2. The predictions for a patient are made out of the following contributions used in each of the plots (Fig. 2). Note that the contributions to the logit are the partial responses. They are referenced to the median value of the variable; that is, the partial response is 0 when the variables are at their median value. P*(death in year 1|x)* = *sigmoid function of (f1(donor_age)* + *f2(ischemic_time)* + *…* + *f9(diagnosis:NICM)* + *f10((recipient_age x diagnosis:ICM))* + *intercept).* In the PRN-Lasso model, there are nine univariate effects and one 2-way interaction (f10). For this patient, P(death in year 1|x) = sigmoid function of the contributing logits listed in Supplementary Table 1 plus the intercept (− 2.335) = − 2.833. Sigmoid (− 2.833) = 0.056. For this patient, the main effect was protective, and the diagnosis was NICM = 1 (nonischemic disease). The only factor that increases risk is donor age 35, but this is small in value. In summary, this patient has protective factors that far outweigh the risk factors, so not surprisingly, the prediction is a good outcome, and this is also the observation. The second patient was 66 years old, had a long ischemic time, infection, and renal failure and was supported by a ventilator pretransplant. This is a high-risk case where 5 risk factors contribute to the mortality risk. Here, the donor age was protective together with the diagnosis. A nomogram that can be used to predict the 1-year probability of death after heart transplantation is shown in Fig. 5.

## Discussion

The development of a new risk calculator for decision support to be used in the clinical care of patients is complex. It is not only about achieving better performance compared to previous decision support tools but also about increasing the understanding and importance of the risk factors involved. To achieve this, the new decision support must be interpretable, which can be difficult when it is based on machine learning. The results from this study show that an interpretable machine learning model is competitive in performance compared to previously developed deep learning models tested on the same data. Although the two IHTSA models have the best overall score in terms of AUCROC, the interpretable PRN-Lasso model was within their confidence intervals, unlike the classical interpretable model, IMPACT. Furthermore, the PRN-Lasso model was better calibrated on the external validation data compared to IHTSA and IMPACT.

Considering the confidence intervals, all the machine learning models in Table 3, PRN-Lasso, EBM, IHTSA and IHTSA recalibrated, are comparable in performance. This supports the view that the limiting factor in overall predictive power is noise; therefore, any model capable of fitting the structure in the data should result in similar performance to the optimum achievable. A particular strength of interpretable machine learning models is the clear link between the input variables and the model prediction.

Why is it important to be able to risk-stratify a patient before transplantation? One reason may be to identify the best combination of risk factors for recipients/donors to optimize the outcome. This also includes avoiding combinations that result in a poor result to make the best use of the available organs^15^. It is also important to understand how the input factors on which the decision support is based arrive at the result. A potential disadvantage of using risk scoring models is that if the model focuses on clinical criteria and is difficult to interpret, any ethically negative effects that are built into the model might not be identified^16^. Furthermore, it can be difficult for a model to identify risk factors from small subgroups. Such negative effects are important to identify early in the development phase^17^. After the model is implemented, it can be difficult to change it. This emphasises the importance of a model being interpretable as well as the importance of evaluation before clinical implementation.

There are currently approximately 15 different algorithms that predict survival after heart transplantation. They all have a relatively poor ability to discriminate i.e. identify an individual patient with increased risk when used on external data^18^. The most cited risk stratification model is the IMPACT score—an interpretable algorithm published in 2011 by Weiss et al.^13^. The scoring model is developed using logistic regression analysis where only recipient characteristics are included. Compared with the IHTSA model developed with artificial neural networks (ANNs), the ability to discriminate the IMPACT score is inferior. On the other hand, IMPACT is easier to interpret than IHTSA. The PRN-Lasso model, which is based on both recipient and donor variables, has the same discriminatory ability as IHTSA and is interpretable as IMPACT. Tree of prediction (TOP), an interpretable model developed using regression trees, showed similar discrimination results in an internal validation, such as PRN-Lasso^19^. However, no external validation is available for this model.

To assess how well decision support can work in clinical practice, it should be appropriately validated. Simple cross-validation is usually not sufficient, and a separate test cohort should be used. This was recently demonstrated in a study evaluating the predictive power of popular ML and statistical algorithms. The authors use different validation techniques to assess the accuracy of the prediction of 1-year mortality. The results showed that temporal validation similar to that used in this study is important due to the temporal changes in the sample of patients and donors^20^.

In medical fields where the patient population is not very large, clinical databases are often required, which gather information from many different centres. Thus, external validation alone is not sufficient, but local validation is equally important. This is due to differences in local processes and protocols that may change the measurement of individual covariates, as well as factors that change over time. The external validation on the Scandia data set shows how well our model trained on UNOS data applies. This indicates that there is a high level of consistency between the measurement of the predictive variables and for the overall mortality trend over time.

In the PRN-Lasso and IHTSA models, the year of transplantation was one of the most significant variables. It is well known that 1-year survival has improved continuously over the last two decades, as have the temporal changes in the sample of patients and donors^20,21^. Interactions between different risk factors may also vary over time, as Hsich et al. recently demonstrated using random forest^2^. If the model does not take this into account, performance will be impaired, as we can see for the IMPACT model in this study. IMPACT was significantly less well calibrated in the external validation cohort than the PRN-Lasso model. The fact that the calibration of the IHTSA models also does not work so well is because the variable is divided into time eras instead of years and the last era in the development cohort was 2010. The PRN-Lasso model treats transplant years as a continuous variable and can therefore extrapolate risk forward in time. The transplant year acts as a recalibration to take into account the gradual decrease in mortality from year to year.

The five most significant variables in the PRN model are also among the top 10 most important variables in the IHTSA model in addition to ischemia^12^. The IMPACT model lacks donor-related variables, which are two of the top five^13^. Both donor age and ischemia are known predictors of 1-year survival. Their efficacy persisted despite strong regularization at the LASSO selection in this study, especially donor age, which has proven to be the most important variable in other prediction models. In the early 1970s, Griepp et al. argued that an ideal heart donor should be younger than 30 years, as confirmed in the present study^22^. A donor age above 30 years carries an increased risk of mortality. Unlike a noninterpretable model, the PRN-Lasso model clearly shows that the risk increase for donor age is not linear but is protective at young ages. Our model also shows that the risk increase caused by ischemia comes at 3 h and not at 4 h, which is an old clinical rule^21^. The fact that ischemia is not ranked as highly in the IHSTA model is probably because this model was not primarily optimised for 1-year survival but for long-term survival time.

When quantifying the univariate and bivariate effects modelled, the input factors whose weights add together to make the final prediction may be checked against clinical expertise. The importance of recipient age for survival varies in the literature. The biological difficulty for the algorithm to model is that the risk of total mortality increases with age, as for other diseases. The risk of transplant-related complications increases with age^2,12^. For example, the ISHLT report shows that mortality risk for 1 year increased after the age of 55^23^. In addition, the risk of rejection is reduced. The immune system is at its most active at younger ages. The findings from this study show an increased mortality rate for older recipients but a protective effect for those between 35 and 55 years of age (Fig. 2a). In contrast to the IHTSA model, we see that the risk again increases for the youngest recipients. However, it is interesting to note that the predicted risk is reduced for younger recipients if the patient is diagnosed with ICM. While this bivariate effect is consistent with crude filtering of the data as noted in the results section, it may be difficult to explain biologically. ICM is traditionally a diagnosis associated with higher mortality, especially compared to NICM. An explanation might be that the aetiology of ICM is often different for younger patients compared to older ones. For an older patient, ICM is usually associated with general atherosclerotic disease, whereas for a younger patient, the cause is different^24^. It may be in the form of a coronary artery anomaly, meaning that the problem is completely bypassed when the heart is replaced.

The results of this study have limitations associated with the retrospective analysis of a registry database, the quality of source data, and the lack of standardization associated with multi-centre studies (such as various immunosuppressive regimens and various matching criteria), as has been described previously^25^. The existence of missing values is another problem that can affect the result. We used a multiple imputation technique to be able to use the entire material and avoid selection bias if one would instead choose to remove patients or variables with missing values. However, this means that the importance of variables with many missing values is more difficult to quantify.

In this study, we present an interpretable sparse algorithm with the same classification performance as today's most well-known deep learning models. In particular, the PRN model is considered to be self-explanatory because the impact of the input variables on the output is transparent. By dividing a complex multivariate algorithm into elements with lower dimensionality, the elements of this additive model are easy to read and can be interpreted by clinicians. Although interpretability is better, the low level of discrimination still persists i.e. low predictive power at the individual level. However, this is a known problem when modelling tabular data where the data are largely preprocessed. Classification performance measured by the AUROC is maintained for the interpretable mode compared with others on the same data. However, the clear weights associated with individual effects enable a detailed discussion to be had about the clinical plausibility of the inputs to the model, which is where the discussion of risk models for high-stakes applications needs to go. This level of transparency provides both a rigorous explanation for predictions made with respect to individual decisions and a diagnostic route for potential failure modes present in the model. Both are essential elements in deciding when the model is and is not safe to use.

## Methods

### Data source

The data set of heart transplant patients was obtained from the United Network for Organ Sharing (UNOS) database, SRTR (Scientific Registry of Transplant Recipients). UNOS (http://www.unos.org) is a nonprofit organization that administers the Organ Procurement and Transplantation Network (OPTN) in the United States of America. The SRTR includes data on all donors, waitlisted candidates, and transplant recipients in the United States submitted by the members of the OPTN. Human error in data entry is minimized by error checks at the time of data entry and internal verification of outliers. The database contains data from October 1, 1987, onwards and includes almost 500 variables that encompass recipient, donor, and transplant information. It consists of both deceased- and living-recipient transplants.

The Ethics Committee for Clinical Research at Lund University, Sweden approved the study protocol (2016/987). The data were anonymized and de-identified prior to analysis, and the institutional review board waived the need for written informed consent from the participants. The research was performed in accordance with the Declaration of Helsinki. No organs/tissues were procured from prisoners.

### Study population

Data on heart donors and the corresponding recipient who were transplanted between January 1, 1987, and June 30, 2020, were collected from the UNOS registry (n = 76,844). Pediatric cases (recipients younger than 18 years, n = 7577), donor age < 15 years and those with incomplete mandatory data (age, sex, duration of follow-up, and/or vital status) were excluded (Fig. 1). The development data set was divided into two temporal cohorts: transplantation performed between 1997 and 2013 (derivation cohort) and after or during 2014 until 2016 (test cohort). The number of variables extracted from the database was 111 in total, where IHTSA uses 43 of them and IMPACT 18. The primary endpoint was 1-year mortality.

To evaluate and calculate the metrics for the models, an external validation cohort was created from SRTR using the same inclusion and exclusion criteria. Here, the endpoint was masked from the development team. The blinded validation set contained patients transplanted between 2017 and 2018, and the latest follow-up was August 31 2020.

The Scandinavian Thoracic Transplantation Database is a registry within the Scandia Transplant organization, incorporating all thoracic transplantations performed in Norway, Denmark, Sweden, Finland, and Estonia (http://www.scandiatransplant.org). The registry has been in existence since 1983 and consists of more than 400 variables, and it is mandatory for all centres performing transplantations. The total number of registered heart transplants was 3930 on December 31, 2020.

### The IMPACT model

IMPACT was created with a data set of heart transplant patients between 1997 and 2008 that was collected from the UNOS database^13^. IMPACT only utilises recipient variables. By apportioning points according to the relative importance of the variables for the 1-year mortality, a risk index was created. The minimum number of scoring points a patient can have is 0, and the maximum is 50. The points are then converted to a predicted probability of 1-year mortality by a formula derived from logistic regression.

### The IHTSA model

The data set used in developing IHTSA was extracted from the ISHLT registry containing HT patients who were transplanted between 1994 and 2010^12^. IHTSA utilises both recipient and donor variables. The survival model consists of a flexible nonlinear generalisation of the standard Cox proportional hazard model. Instead of using a single prediction model, this model integrates ensembles (10 submodels) of ANNs. Each ANN consisted of an input layer (43 inputs), one hidden layer (18 nodes) and an output layer. In addition, its prediction capability is not limited to 1 year. The variables hypertension and antiarrhythmic drugs are not recorded in the UNOS database from 2007 onward. To handle this when evaluating the IHTSA model, we imputed those two variables with random values taken from the earlier time period. In the recalibrated IHTSA model, these two variables were excluded, and the neural network retrained (calibrated), utilizing a fivefold cross-validation of the patients between 1997 and 2008 in UNOS. The same training procedure was used as described in the original IHTSA article, but we did not carry out any new variable selection. We called this model the recalibrated IHTSA model^26^.

### Development of the partial response network (PRN) and PRN-Lasso model

The PRN is probabilistic; hence, it readily manages data imbalance by adjusting the threshold for class assignment, and it models the logarithm of the predicted odds ratio using a linear combination of terms. This is similar to logistic regression, except that the additive elements are semiparametric; hence, they are nonlinear functions of the original variables. The modeling methodology is explained in the “Methods” section and in the supplementary Methods. Formally, the PRN has the structure of a GAM, so the interpretation of the model is the model itself. Each input contributes an amount to the model prediction, given by explicit functions of only one or two variables.

An overview of the stages involved in developing the model is as follows. First, a multilayer perceptron (MLP) is fitted to the data. This pretrained network provides the initial estimate of the posterior probability of class membership, P(Class|x). However, it has the form of a black box. Second, we carry out a functional ANOVA decomposition of the logit(P(Class|x)) anchored at the median of the data. This results in a set of partial responses of one and two variables, together with orthogonal functions involving more than two variables, which, together, add up to exactly the predictions of the logit of the MLP.

The third step is to take the univariate and bivariate partial responses only, hence truncating the ANOVA decomposition. To recalibrate the model, these partial responses form the inputs for a GAM, which is the logistic regression Lasso10. Lasso optimisation is particularly efficient for model selection, as it applies L1 regularization to collapse the coefficients of the less informative inputs to zero. The result of this step is a fully interpretable model in the form of a GAM with main effects and pairwise interactions derived from the original black box, the MLP.

**Figure 6:**
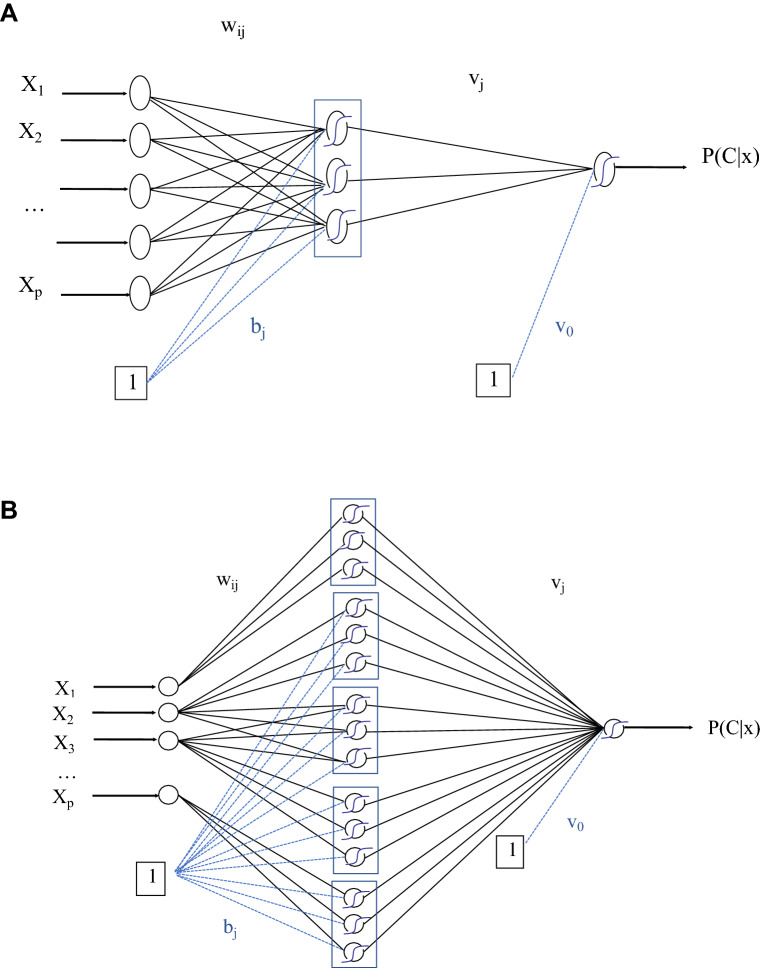
Structure of the original fully connected MLP (top) and the partial response network (PRN) (bottom), which has the structure of a self-explaining neural network. The structure of the partial response network (PRN) is comprised of modular replicas of the relevant weights from the original multilayer perceptron (MLP) for each univariate or bivariate response retained by the Lasso, adjusted by Eqs. (6–11) to initialize the PRN with the exact functional response of the Lasso. Further training smooths out the responses and improves predictive performance. The equations can be found in the supplementary material.

However, the presence of uninformative variables in the original MLP may reduce performance since they will affect the response of the variables retained in the final model selection. For this reason, the three steps are repeated with a new model structure to optimise performance. In step 4, the previously derived GAM is mapped onto the structure of a Generalised Additive Neural Network (GANN). The GANN is then further trained by backpropagation, so its classification performance is optimized, resulting in what we call the PRN. This new network is shown in Fig. 6.

Finally, the same process of ANOVA decomposition followed by Lasso modelling can be applied to the output of the PRN rather than the MLP. The result is the PRN-Lasso. In all of the figures, partial responses derived from the MLP are shown as dashed lines and those from the PRN using solid lines. The performance of the PRN and PRN-Lasso models are listed in the Results section.

### Statistical analysis

Statistical analyses were performed using the Stata MP statistical package version 16.1 (2021) (StataCorp LP, College Station, TX). Data are presented as the means with standard deviation, median with interquartile range (IQR) and frequency as appropriate. Unpaired Mann–Whitney U-tests were used to compare continuous variables, and χ2 tests were used to compare categorical variables among groups. The Hosmer–Lemeshow goodness-of-fit test was used to assess predictive accuracy. The discriminatory power for 1-year mortality was assessed by calculating the area under the receiver operating curve (AUROC). To compare different areas, the nonparametric approach described by DeLong was used^27^.

We used a multiple imputation technique consisting of probability imputation with stratification of the era. Each missing value was imputed 10 times with a random existing data point from another patient, resulting in 10 study cohorts with a variation in variables that had missing data. We combined all imputed datasets into the derivation cohort [DC], consisting of the 10× imputed datasets (n = 313,150), resulting in a counterweight of random fluctuations. Note that the overall proportion missing of all data points for our PRN-Lasso model was < 1% (0.69%), which is significantly less than the Impact and IHTSA models. The number of complete cases is noted in Table 1.

## Supplementary Information

Supplementary Information.

## Data Availability

The data that support the findings of this study are available from the SRTR (https://www.srtr.org/requesting-srtr-data/data-requests/), but restrictions apply to the availability of these data, which were used under license for the current study and are not publicly available. Data are however available from the authors upon reasonable request and with permission of SRTR.

## Code availability

The PRN Lasso model was developed in MATLAB 2020b (MathWorks, Natick, MA). The code files are available on request from the authors.
