# Supplementary material for: Enhanced survival prediction using explainable artificial intelligence in heart transplantation
Source: Sci Rep. 2022 Nov 14;12:19525. doi: 10.1038/s41598-022-23817-2 (PMC9663731; doi:10.1038/s41598-022-23817-2)
Supplement: Supplementary file 1 — Supplementary Information. [file 41598_2022_23817_MOESM1_ESM.pdf]

Supplementary Information for

Enhanced survival prediction using explainable artificial intelligence in heart transplantation

Paulo J. G. Lisboa, Manoj Jayabalan, Sandra Ortega-Martorell, Ivan Olier, Dennis Medved, Johan Nilsson

Supplementary Methods

The Partial Response Network (PRN) and PRN-Lasso model

The PRN is built in a stepwise manner as follows.

1. Pre-trained MLP

The first step is to train an MLP to estimate the posterior distribution of membership of class *C*, *P(C|x).* The predictions of this model are then transformed by inverting the sigmoid function at the output node, to obtain the *logit(P(C|x))* which is the logarithm of the odds-ratio of the posterior probability.

2. ANOVA decomposition

The key step in the proposed method is to express the multivariate function consisting of the model predictions transformed into the *logit(P(c|x)),* by application of the ANOVA decomposition shown in (1) which is anchored at the overall median of the data[1] and is equal to the summation of component variables phi_i, phi_ij, etc. which are defined below:

$$\begin{aligned} \boldsymbol{logit}(\boldsymbol{P}(\boldsymbol{C}|\boldsymbol{x})) \equiv \boldsymbol{\varphi}(\mathbf{0}) &+ \sum_{i} \boldsymbol{\varphi}_{i}(\boldsymbol{x}_{i}) \\ &+ \sum_{i \neq j} \boldsymbol{\varphi}_{ij}(\boldsymbol{x}_{i}, \boldsymbol{x}_{j}) + \cdots \\ &+ \sum_{i_1 \neq \cdots \neq i_d} \boldsymbol{\varphi}_{\,i_1 \ldots i_d}(\boldsymbol{x}_{i_1}, \ldots, \boldsymbol{x}_{i_d})\,. \end{aligned} \quad (1)$$

The terms in the summation are given by:

$$\boldsymbol{\varphi}(\mathbf{0}) = \boldsymbol{logit}(\boldsymbol{P}(\boldsymbol{C}|\mathbf{0})) \quad (2)$$

$$\boldsymbol{\varphi}_{i}(\boldsymbol{x}_{i}) = \boldsymbol{logit}\left(\boldsymbol{P}(\boldsymbol{C}|(\mathbf{0}, .., \boldsymbol{x}_{i}, .., \mathbf{0}))\right) - \boldsymbol{\varphi}(\mathbf{0}) \quad (3)$$

$$\begin{aligned} &\boldsymbol{\varphi}_{ij}(\boldsymbol{x}_{i}, \boldsymbol{x}_{j}) = \\ &\boldsymbol{logit}\left(\boldsymbol{P}\left(\boldsymbol{C}|(\mathbf{0}, .., \boldsymbol{x}_{i}, .., \boldsymbol{x}_{j}, .. \mathbf{0})\right)\right) - \boldsymbol{\varphi}_{i}(\boldsymbol{x}_{i}) - \\ &\boldsymbol{\varphi}_{j}(\boldsymbol{x}_{j}) - \boldsymbol{\varphi}(\mathbf{0}). \end{aligned} \quad (4)$$

The general form of the terms in (1) is a recursive function of nested subsets of the covariate indices $\{i_1, \ldots, i_n\}$:

$$\begin{aligned} &\boldsymbol{\varphi}_{\,i_1 \ldots i_n}(\boldsymbol{x}_{i_1}, \ldots, \boldsymbol{x}_{i_n}) = \boldsymbol{logit}\left(\boldsymbol{P}(\boldsymbol{C}|\boldsymbol{x}_{i_1}, \ldots, \boldsymbol{x}_{i_n})\right) - \\ &\textstyle\sum_{\{i_1 \neq \cdots \neq i_{n-1}\}} \boldsymbol{\varphi}_{\,i_1 \ldots i_{n-1}}(\boldsymbol{x}_{i_1}, \ldots, \boldsymbol{x}_{i_{n-1}}) - \boldsymbol{\varphi}(\mathbf{0}). \end{aligned} \quad (5)$$

Recall that the standardisation of data is such that $\boldsymbol{x}_{i} = 0 \leftarrow median(\boldsymbol{x}_{i})$, which sets the overall median of the data as the reference point for the calculation of the logit function, that is to say, the point at which all of the variables take the value 0 and where, by construction, all of the partial responses also vanish, leaving only the constant term $\boldsymbol{\varphi}(\mathbf{0})$.

Two things to note: expression (1) is not an equation but an identity involving a finite number of terms, 2^P where *P* is the number of input variables in the MLP; and the component functions $\boldsymbol{\varphi}_{\,i_1 \ldots i_n}(\boldsymbol{x}_{i_1}, \ldots, \boldsymbol{x}_{i_n})$, which we call partial responses, are calculated directly from the predictions of the MLP via the logit transformation. The form of the partial responses defined in (2)-(4) is designed to ensure orthogonality, so that each term in (1) comprises additional information. This can be seen from the form of the generic term in (5).

While the main effects, which are the univariate terms in (3), are numerically identical to the partial dependency plots often used to visualise the operation of non-linear multivariate models, the bivariate effects in (4) are not simply cuts through the model response function, but have the univariate terms removed, so that $\boldsymbol{\varphi_{ij}}(\boldsymbol{x_i}, \boldsymbol{x_j} = 0) = \boldsymbol{\varphi_{ij}}(\boldsymbol{x_i} = 0, \boldsymbol{x_j}) = 0$. This denotes the orthogonality property under the Dirac measure, which is a special case of the general formulation of the terms of the ANOVA decomposition for a given function f(x), namely $\varphi_S(x_S) = \int f(x) d\mu(x_S) - \Sigma_{T \subset S} \varphi_T(x_T)$ where S indicates the set of variables in term φ_S and $\mu(x_S)$ is the chosen measure. In the case of the Dirac measure, $\mu(x_S) = \delta(x - x_S)$ meaning that the function f(x) is evaluated at the anchor point $x = x_S$.

3. Lasso optimisation

The third step in the proposed method is to retain, from the ANOVA decomposition of the logit of the pre-trained classifier, only the terms that involve just one or two variables. This amounts to truncating the decomposition (1) followed by a re-calibration.

The terms defined by (3) and (4) become the input variables in a linear model which is logistic regression regularised by the Least Absolute Shrinkage and Selection Operator (Lasso) 10. This is a powerful feature selection method that scales well for a large number of variables. It uses L1 regularisation to carry out feature selection, which slides the coefficients gradually to zero resulting in a sparse model.

This optimisation is computationally feasible because there are in total $P * (P + 1)/2 \ll 2^P$ univariate and bivariate effects, which is quadratic in the input dimensions of the original data, rather than exponential as the number of terms in (1).

The resulting model has a logit function consisting of partial responses added together. This is the equivalent of the score index in logistic regression, which is the sum $\beta_0 + \sum_i \beta_i x_i$ taken over the inputs $\boldsymbol{x_i}$: $i = 1..P$. In our case, the input values have been replaced by non-linear functions of the variables, each function involving one or two variables at a time in order to ensure interpretability. As these functions sum together to make the model prediction, in a truncated ANOVA decomposition (1), the variables directly relate to the predicted outcome and the final model can be represented with a nomogram.

4. The PRN model

The application of the Lasso to generate a sparse model has the effect of removing the least informative input variables, improving the signal to noise ratio of the truncated ANOVA decomposition compared with the multivariate function defined by the logit of the predictions of the original MLP. It is now possible to refine the form of the partial responses, by mapping the Lasso model into the form of a GANN. This is initialised to exactly replicate the output of the Lasso model. This mapping is done in order to enable training to continue by back-propagation to fully optimise the partial responses.

This new network is shown in Fig. 6. It implements univariate and bivariate functions with a modular structure. The weight mappings to reproduce the Lasso outputs are given below, following the notation in Fig. 6 i.e. hidden node weights denoted by wij and output weights by vj:

i. Univariate partial response corresponding to input X_i

   This is shown for input X_I. The hidden layer weights w_{Ij} connected to node X_I are the same as in the original MLP but the weights and bias to the output node need to be adjusted as follows:

$$v_j \rightarrow \beta_i * v_j \tag{6}$$

$$v_0 \rightarrow \beta_i * \left(v_0 - logit\big(P(C|0)\big)\right) \tag{7}$$

ii. Bivariate partial response for input pair $\{X_k, X_l\}$

This is illustrated in Fig. 6 for inputs X_2 and X_3. This time, in order to replicate the partial response multiplied by the Lasso coefficient, it is necessary to add three elements to the structure, namely, a univariate partial response for each of the inputs involved and a coupled network that both inputs feed into together. We will use the generic input indices *k* and *l* to avoid confusion with the hidden node index *j*. The hidden layer weights once again remain unchanged from the original MLP. The output layer weights and bias for the network structure representing the univariate term associated with input *k* (and similarly for input *l*) are adjusted by

$$v_j \rightarrow (\beta_k - \beta_{kl}) * v_j \tag{8}$$

$$v_0 \rightarrow (\beta_k - \beta_{kl}) * \left(v_0 - logit\big(P(C|0)\big)\right) \tag{9}$$

whereas the weights and bias for the coupled network are changed according to:

$$v_j \rightarrow \beta_{kl} * v_j \tag{10}$$

$$v_0 \rightarrow \beta_{kl} * \left(v_0 - logit\big(P(C|0)\big)\right). \tag{11}$$

iii. Finally, the intercept of the logistic Lasso, β_0 is also added to the output node bias v_0.

An interesting property of this mapping is that the module for a bivariate term involves a combination of univariate and bivariate functions. This means that following further training, what started as an interaction term may result in two independent univariate effects, even if they were not present in the Lasso.

5. PRN-Lasso

Following optimisation of the PRN, the orthogonality property of the partial response terms will have been broken. It is possible to retrieve this property by repeating steps 2-3 but applied to the outputs of the PRN rather than those of the original MLP. This results in a set of refined partial responses, suitably re-calibrated by the Lasso, which is the PRN-Lasso model.

In all of the figures, partial responses derived from the MLP are shown as dashed lines and those from the PRN using solid lines.

References

1. Lisboa PJ, Etchells TA, Jarman IH, et al. Partial logistic artificial neural network for competing risks regularized with automatic relevance determination. *IEEE Trans Neural Netw* 2009; **20**(9): 1403-16.

Supplementary Figures

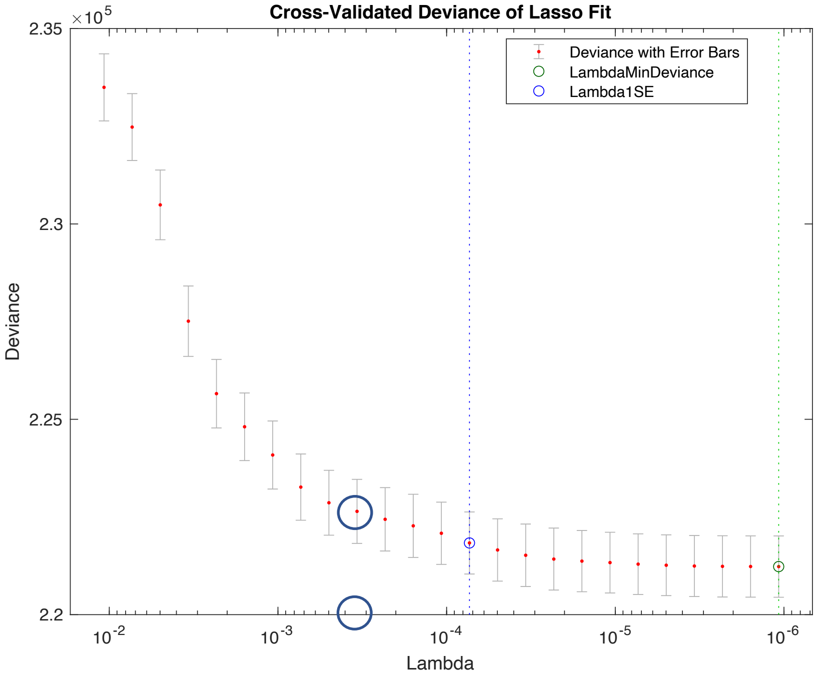

Supplementary Fig. 1 First Lasso fit showing the Deviance i.e. -2*log-likelihood and corresponding values of the regularisation parameter λ.

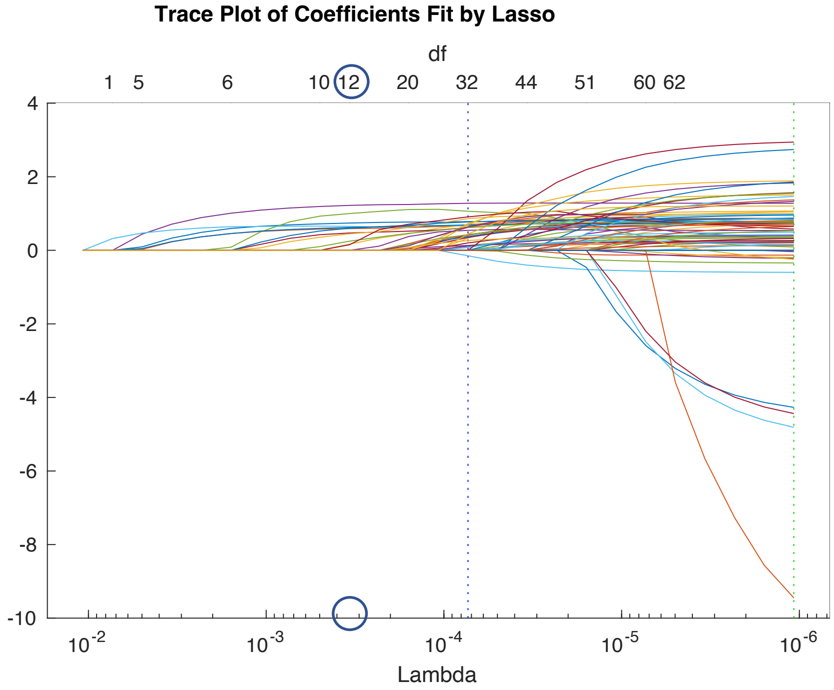

Supplementary Fig. 2 First Lasso models showing the actual β values for each regularisation parameter λ. The number of degrees of freedom is listed at the top.

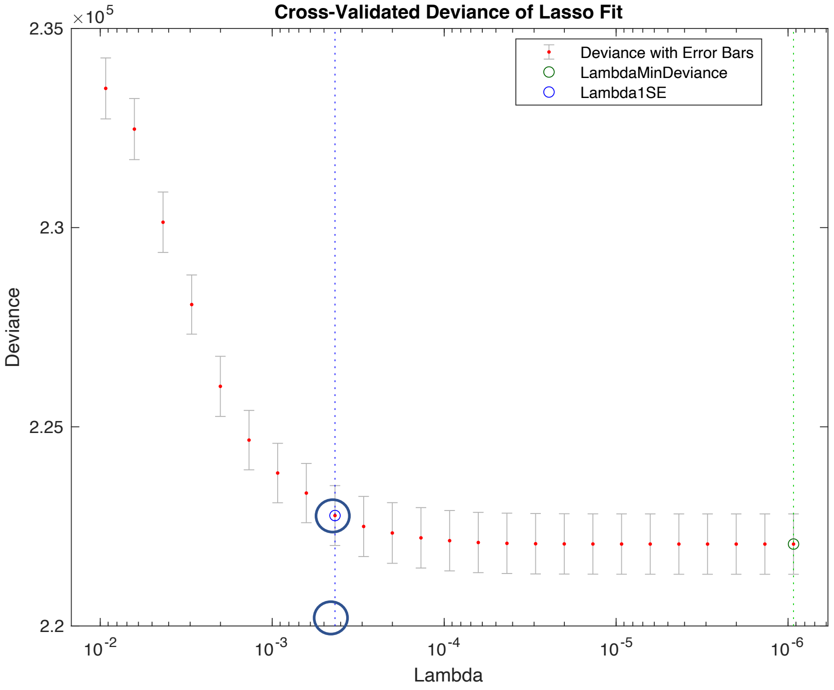

Supplementary Fig. 3 Second Lasso fit showing the Deviance and corresponding values of the regularisation parameter λ.

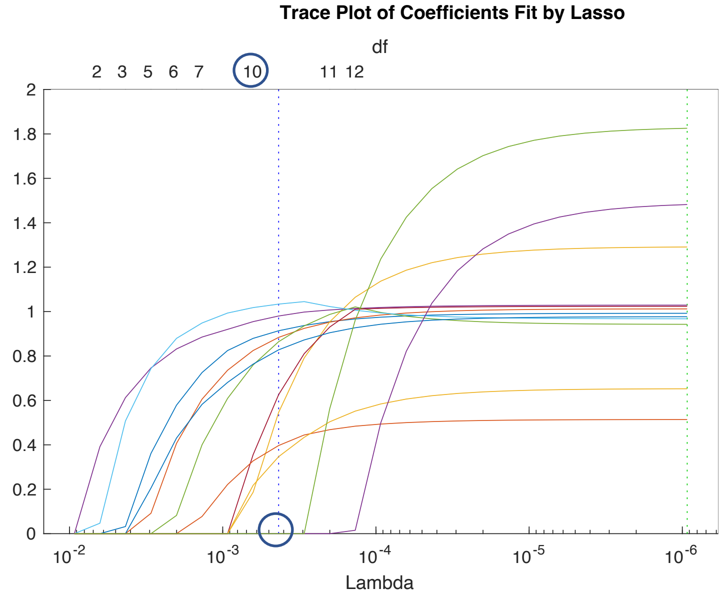

Supplementary Fig. 4 Second Lasso models showing the actual β values for each regularisation parameter λ. The number of degrees of freedom is listed at the top.

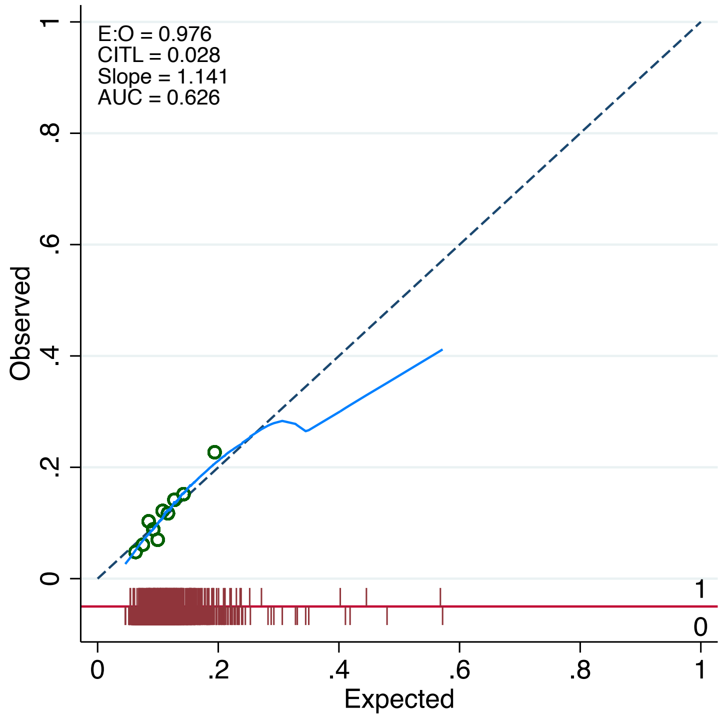

Supplementary Fig. 5 The calibration plot of the PRN-Lasso model shows observed against expected probabilities for assessment of the prediction model with the overall Scandia Thoracic Transplantation Database full data set imputed validation cohort (n=2,293) according to the TRIPOD guidelines. The red spike plot shows the distribution of events and non-events. Green circles the expected probabilities in groups with 95% CI. The green line demonstrates the lowess smother. The model performance statistics are summarised with EO: exp/obs ratio; CITL: calibration-in-the-large; and AUC: area under the curve.

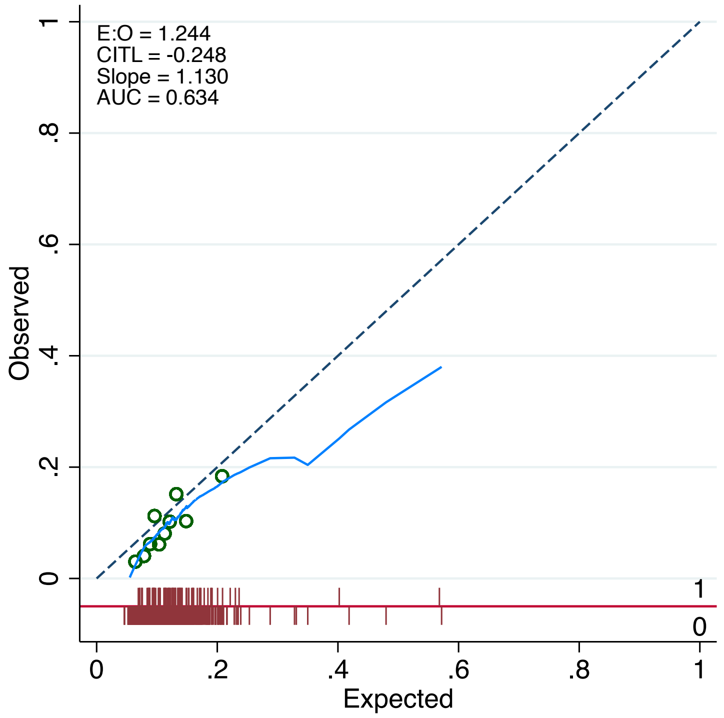

Supplementary Fig. 6 The calibration plot of the PRN-Lasso model shows observed against expected probabilities for assessment of the prediction model with the overall Scandia Thoracic Transplantation Database complete data set with no missing values validation cohort (n=982) according to the TRIPOD guidelines. The red spike plot shows the distribution of events and non-events. Green circles the expected probabilities in groups with 95% CI. The green line demonstrates the lowess smother. The model performance statistics are summarised with EO: exp/obs ratio; CITL: calibration-in-the-large; and AUC: area under the curve.

Supplementary Tables

Supplementary Table 1. Case 1.

Variable	Value	Contributing logit	Beta coef.	Effect	Individual Odds
Donor age (yrs)	35	0.075	0.914	0.069	1.07
Ischemic time (min)	99	-0.065	0.884	-0.057	0.94
Recipient age (yrs)	42	-0.025	0.546	-0.014	0.99
Creatinine (µmol/l)	88	-0.076	0.980	-0.074	0.93
Infection within 2 weeks	0	0	0.864	0	1.00
Ventilator at OHT	0	0	1.034	0	1.00
Female gender (recipient)	0	0	0.626	0	1.00
Transplant era (year)	2017	-0.358	0.828	-0.296	0.74
Diagnosis: NICM	NICM	-0.31	0.397	-0.125	0.88
Recipient age (yrs) * Diagnosis: ICM		0	0.347	0	1.00
Intercept		-2.335		-2.335	
Effect				-2.833	
One mortality risk				5.6%	

Supplementary Table 2. Case 2.

Variable	Value	Contributing logit	beta	Total effect	Individual Odds
Donor age (yrs)	26	-0.053	0.914	-0.048	0.95
Ischemic time (min)	214	0.086	0.884	0.076	1.08
Recipient age (yrs)	66	0.128	0.546	0.070	1.07
Creatinine (µmol/l)	194	0.594	0.980	0.582	1.79
Infection within 2 weeks	1	0.409	0.864	0.354	1.42
Ventilator at OHT	1	1.108	1.034	1.145	3.14
Female gender (recipient)	0	0	0.626	0	1.00
Transplant era (year)	2018	-0.376	0.828	-0.311	0.73
Diagnosis: NICM	NICM	-0.315	0.397	-0.125	0.88
Recipient age (yrs) * Diagnosis: ICM		0	0	0.347	0
Intercept		-2.335		-2.335	
Effect				-0.592	
One mortality risk				35.6%	
